# Prevalence of peste des petits ruminants virus antibodies in sheep and goats sera from Central-Western Sudan

**DOI:** 10.4102/ojvr.v90i1.2074

**Published:** 2023-02-28

**Authors:** Safa E.M. Ali, Yassin A.M. Ahmed, Alwia A. Osman, Omiema A. Gamal Eldin, Nussieba A. Osman

**Affiliations:** 1 Department of Pathology, Parasitology and Microbiology, College of Veterinary Medicine, Sudan University of Science and Technology, Khartoum-North, Sudan; 2 Equine-Specialty Center, Global Veterinary Services and Agriculture, Doha City, Qatar; 3 Nokhbat Alnawadir Veterinary Pharmacy, Al Duwadimi, Saudi Arabia; 4 General Directorate of Animal Health & Epizootics Diseases Control, Ministry of Animal Resources, Khartoum, Sudan

**Keywords:** peste des petits ruminants, PPR, PPRV, Sudan, sheep, goats, antibodies, seroprevalence

## Abstract

Clinical signs suggestive of peste des petits ruminants (PPR) involved herds of small ruminants, which were described elsewhere in Sudan. Peste des petits ruminants was confirmed using an Immunocapture ELISA (IC-ELISA) assay in samples of infected and dead animals in areas of outbreaks. Therefore, to update information regarding the current situation and for assessment of the serological prevalence of PPR in small ruminants mingled at Central and Western Sudan during 2018–2019, 368 sera were collected from sheep (325 sera) and goats (43 sera) with different ages and breeds. These sera included 186 sera (173 sheep and 13 goats) from White Nile State and 182 sera (152 sheep and 30 goats) from Kordofan States. Competitive ELISA demonstrated higher prevalence of PPRV antibodies of 88.9%, 90.7% and 88.6% in both sheep and goats, goats, and sheep sera, respectively. Moreover, 100%, 94.7% and 78.5% seroprevalence values were demonstrated in South Kordofan, North Kordofan and White Nile States. The higher seroprevalence values detected in sera of unvaccinated sheep and goats indicated the wide exposure of these animals to PPRV and presence of protection following PPR viral infection. The findings of the study indicated that PPR is endemic in the surveyed areas of Sudan.

**Contribution:**

The study will contribute effectively to the global eradication programme of PPR organised by the World Organization for Animal Health (WOAH, formerly OIE) and Food and Agriculture Organization (FAO). To completely eliminate PPR from Sudan by 2030, local efforts should be directed towards effectively and wholly vaccinating small ruminants using PPRV vaccine especially in routes of seasonal animal’s movement and shared grazing areas.

## Introduction

Peste des petits ruminants (PPR), also known as sheep and goat plague, is an acute, highly contagious and fatal viral disease causing high mortality rates in naïve populations of domestic small ruminants and some wildlife species (Diallo & Libeau 2014; Office International des Epizooties [OIE] 2021). In endemic regions, the economic is seriously affected by the presence of PPR through limiting the trade, export and import of new animal breeds, the development of intensive livestock production and availability of protein for human consumption (Banyard et al. 2010; Singh et al. 2014).

Peste des petits ruminants is caused by peste des petits ruminant virus (PPRV), a member of the *small ruminant morbillivirus* species in the *Morbillivirus* genus of the Paramyxoviridae family (Amarasinghe et al. 2019; Maes et al. 2019).

Pest des petits ruminants, a fatal disease of goats with high mortality, was first described in the Ivory Coast (Cote d’Ivoire) in West Africa (Gargadennec & Lalanne 1942). Peste des petits ruminants affects mainly lambs and kids causing severe mortality (Ozkul et al. 2002). Depending upon the extent of the predisposing factors and virulence of the virus strain, the severity of the disease can vary as peracute, acute and subacute; however, usually the course of PPR runs as an acute (Braide 1981; Kulkarni et al. 1996; Obi et al. 1983; OIE 2021).

In the early 1970s, the first outbreak of the disease in sheep and goats was in South Gadaref, Eastern Sudan (Elhag Ali 1973). Based on the clinical signs appeared in the affected animals, the disease was first misdiagnosed as Rinderpest (RP); however, later PPRVs were isolated and the existence of PPR in the country was documented (Elhag Ali & Taylor 1984). Subsequently, outbreaks of the disease were reported in two areas in Central Sudan; Sinnar in 1971 and Mieliq in 1972 (Elhag Ali & Taylor 1984). During the 1990s, PPR outbreaks continued to occur in sheep and goats in Central Sudan, specifically in Gezira State (El Hassan et al. 1994) and Khartoum State (Zeidan 1994). Serological surveys of PPR performed among small ruminants indicated existence of the disease in all areas investigated (Abdalla et al. 2012; El Amin & Hassan 1999; Haroun et al. 2002; Ishag et al. 2015; Osman et al. 2009, 2018; Rasheed 1992; Saeed et al. 2010, 2017; Saeed, Abdel-Aziz & Gumaa 2018; Salih et al. 2014; Shuaib et al. 2014). Currently, PPR is endemic in the country with outbreaks occurring regularly in small ruminants leading to significant economic losses (Osman et al. 2018; Saeed et al. 2017).

In Sudan, at first sheep and goats were known as the only hosts of PPR. However, lately camels and gazelles were described as possible unusual hosts (Asil et al. 2019; Khalafalla et al. 2010). In sheep and goat populations with high risk of PPR viral infection, the disease is controlled by focused vaccinations followed by mass vaccination campaigns. In Sudan, the live-attenuated PPR vaccine originated from Nigeria 75/1 strain (Diallo et al. 1989) is produced locally for vaccination of small ruminants in the field on yearly basis (Fadol & El Hussein 2004). Despite implementation of the vaccination programme, PPR outbreaks were constantly reported in Sudan, on an annual basis, indicating persistence of the disease. This might be due to application of the PPR vaccine in restricted areas in the country.

**FIGURE 1:**
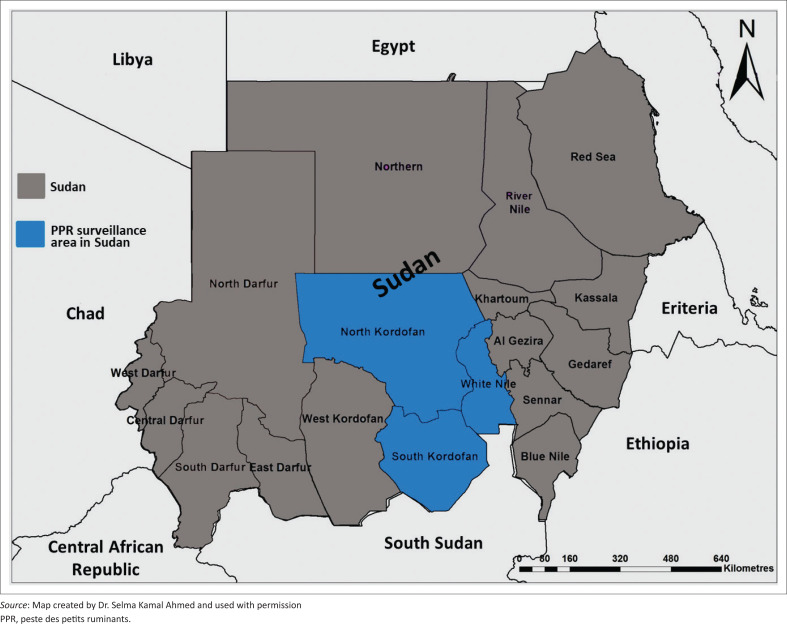
Locations of serological investigations of peste des petits ruminants in White Nile and Kordofan States, Sudan.

In response to several suggestive PPR outbreaks occurred lately in the country, the study was designed to update information regarding the current situation, investigate the presence and determine the prevalence of PPRV antibodies among sheep and goat flocks mingled at the Central (White Nile State) and Western (Kordofan States) Sudan (Figure 1). These were accomplished by serological detection of antibodies against PPRV in sera of small ruminants from these areas.

## Materials and methods

### Study area

#### White Nile State

**FIGURE 2:**
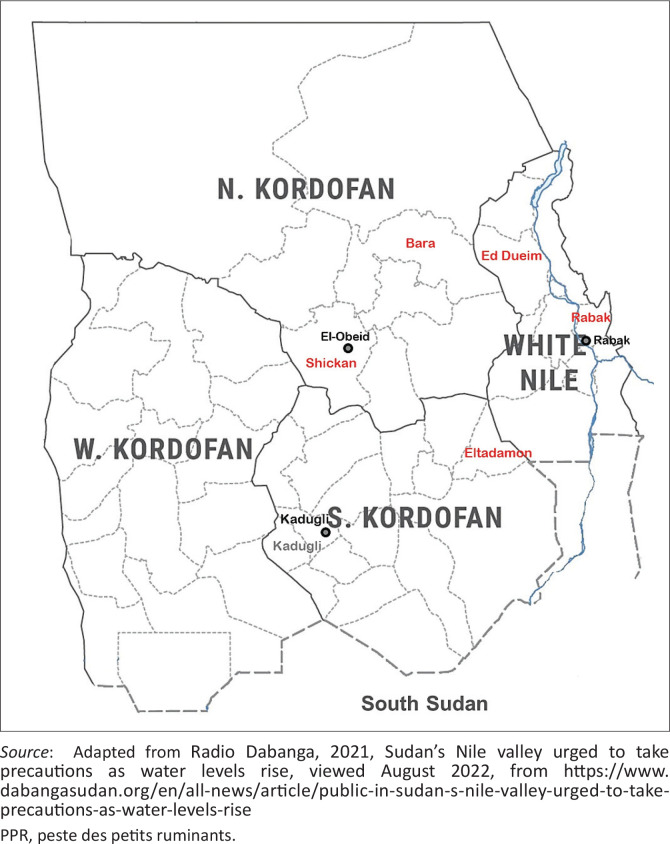
Districts and localities of serological investigations of peste des petits ruminants in White Nile, North Kordofan and South Kordofan States. State capitals are presented.

White Nile State, situated in the central area of Sudan (Figure 1), has an area of 39 701 km^2^ (2008 estimate). Rabak is the capital of the state. It is administratively subdivided into four districts, ‘Ed Dueim, Al Gutaina, Kosti and Al Jabalien’, which is subdivided into nine localities: ‘Ed Dueim, Al Gutaina, Kosti, Rabak, Al Jabalien, Tendulti, Um Remta, Alsalaam and Guli’ (Figure 2). White Nile State is bordered by Khartoum, Gezira and Sinnar States in the East, North Kordofan (NK) in the North, South Kordofan (SK) in the West and the Republic of South Sudan (RSS) in the South.

#### Kordofan States

Kordofan (Kurdufan) States, which covers an area of 376 145 km^2^ (146 932 miles^2^) (2011 estimate), is composed of three states: ‘North-, South- and West Kordofan’ (Figure 1). It is largely an undulating plain, with the Nuba Mountains in the southeast quarter. During the dry season, the area is desert but it is fertile during the rainy season from June to September.

North Kordofan (NK) has an area of 185 302 km^2^ and consists mostly of large grazing areas. El-Obeid is the capital of the state. It is divided into eight localities ‘Sodari, Jabrat El Sheikh, Bara, West Bara, Umm Dam, Shickan, El Rahad and Om Ruwaba’ (Figure 2). North Kordofan is bordered by Northern State in the North, North Darfur in the West, West and South Kordofan in the South, and Khartoum and White Nile in the East.

South Kordofan (SK), centred on the Nuba Hills, has an area of 158 355 km^2^. The state capital is Kadugli. South Kordofan is divided into 5 areas, ‘Dilling, Rashad, Abu Gebaiha, Talodi and Kadugli’, which are further subdivided into 16 localities ‘Al Qoz, Dilling, Habila, Rashad, Alabasia, Eltadamon, Delami, Abu Kershola, Kadogli, Heiban, Um Derin, Buram, Talodi, Ghdeer, Abu Gebaiha and Al Leri’. South Kordofan is bordered by NK in the North, West Kordofan in the West, White Nile in the East, and RSS in the East and the South (Figure 2).

### Sample collection and preparation

During 2018–2019, a total of 368 sera from sheep (325 sera) and goats (43 sera) were collected from three states of Sudan, namely White Nile (Central Sudan), NK and SK (Western Sudan) (Table 1, Figure 1).

**TABLE 1:** Sheep and goat sera collected from White Nile and Kordofan States in Sudan during 2018–2019.

Date of collection	District/locality	State	Animal species		Total number of sera
			Sheep	Goats	
October 2018	Ead Aljam, Ed Dueim locality	White Nile State	92	10	102
October 2018	Gezira Um Jaar, Ed Dueim locality	White Nile State	44	3	47
November 2018	Gezira Aba, Rabak locality	White Nile State	37	0	37
Total			173	13	186
February 2018	Para, Para locality	North Kordofan State	0	9	9
February 2018	Elobied, Shickan locality	North Kordofan State	10	0	10
January 2019	Altartar, Eltadamon locality	South Kordofan State	142	21	163
Total			152	30	182
Total number of sera	-	-	325	43	368

A total of 186 sera from sheep (*n* = 173) and goats (*n* = 13) were collected from three districts in Ed Dueim and Rabak localities of the White Nile State in Sudan during October 2018 – November 2018. These sera were collected from Ead Aljam, Ed Dueim locality (102 sera, 92 sheep and 10 goats); Gezira Um Jaar, Ed Dueim locality (47 sera, 44 sheep and 3 goats) and Gezira Aba, Rabak locality (37 sheep sera) (Table 1, Figure 2).

In addition, a total of 182 sera from sheep (*n* = 152) and goats (*n* = 30) were collected from NK and SK States in Western Sudan during 2018 and 2019. During 2018, 19 sheep and goat sera were collected from two districts in two localities: Para, Para locality (9 goat sera) and Elobied, Shickan locality (10 sheep sera) in NK State. During 2019, 163 sera from sheep (n = 142) and goats (n = 21) were collected from Altartar districts in Eltadamon locality, SK State (Table 1, Figure 2).

These sera were originated from sedentary uninfected, nonvaccinated sheep and goats, above one year of age and of different local breeds. All sera were collected from private farms in which permissions were obtained from animal owners before initiation of sample collection. Of note, animal owners declared the complete absence of any vaccination practices against PPR in sheep and goat herds in these areas.

Blood samples were collected aseptically from animals, and sera were prepared following the standard procedure as described previously (Osman et al. 2018) and stored at −20 °C.

### Competitive-enzyme linked immunosorbent assay

A competitive screening ELISA kit ‘ID Screen^®^ PPR Competition kit for the detection of antibodies against PPR in sheep and goats serum and plasma’ developed by CIRAD-EMVT, FAO reference laboratory for PPR in Montpellier, France (IDVet Innovative Diagnostics, France), was used for testing of these sera following the manufacturer’s instructions.

### Ethical considerations

An approval to conduct the study was obtained from the Sudan University of Science and Technology Institutional Ethics Committee (DSR – IEC – 02-2-2017).

## Results

### Description of the clinical disease in sheep and goats and outbreaks investigations

Between October 2018 and November 2018, outbreaks suggestive of PPR involved sheep and goats in three different districts in two localities, Ead Aljam in Ed Dueim locality, Gezira Um Jaar in Ed Dueim locality and Gezira Aba in Rabak locality, of the White Nile State (Central Sudan) (Figure 2).

During 2018, two outbreaks suggestive of PPR occurred mainly in sheep and goats and were reported in two districts in two different localities, Para in Para locality and Elobied in Shickan locality, of the NK State (Western Sudan). In 2019, another two outbreaks suggestive of PPR were reported in one district, Altartar in Eltadamon locality, of the SK State (Western Sudan) (Figure 2).

In all these outbreaks, both infected sheep and goats showed clinical signs described normally in suspected cases of PPR including dullness, depression, dry muzzle, sudden onset of pyrexia, anorexia, nasal and ocular discharges, mouth lesions, stomatitis, emaciation and diarrhoea. Respiratory signs, dyspnea, rapid and laboured breathing associated with pneumonia were also observed. These cases were associated with higher morbidity and mortality rates and in some cases preceded to sudden death which was mostly observed among young animals.

For confirmation of the clinical diagnosis, samples from infected and dead animals were collected by the veterinary authorities in areas of outbreaks. Peste des petits ruminants was confirmed by testing these samples at the Central Veterinary Research Laboratory (CVRL), Soba, Khartoum, using an Immunocapture ELISA (IC-ELISA) assay. After PPR had been diagnosed and confirmed as the leading cause of these outbreaks, a mass vaccination campaign was undertaken by the local veterinary authorities in these states, in order to control the disease.

### The overall seroprevalence of peste des petits ruminants virus antibodies in both sheep and goat sera

As demonstrated by the C-ELISA assay, 327/368 of the tested sheep and goat sera were positive with 88.9% overall seroprevalence of PPRV antibodies. On the species basis, the higher overall seroprevalence of PPRV antibodies was observed among goats (90.7%, 39/43 sera) rather than sheep (88.6%, 288/325 sera) (Table 2).

**TABLE 2:** The overall seroprevalence of peste des petits ruminants virus antibodies, in sheep and goat sera, as demonstrated by Competitive-enzyme linked immunosorbent assay.

Animal species	Total number of sera	%	Number positive	%	Number negative	%
Sheep	325	88.3	288	88.6	37	11.4
Goats	43	11.7	39	90.7	4	9.3
Total number of sera	368	100.0	327	88.9	41	11.1

### The overall seroprevalence of peste des petits ruminants virus antibodies in both sheep and goat sera in surveyed states of Sudan

Within the surveyed States of Sudan, the highest overall seroprevalence of PPRV antibodies in both sheep and goat sera was demonstrated in SK State (100%, 163/163), followed by NK State (94.7%, 18/19) and the least seroprevalence was detected in the White Nile State (78.5%, 146/186) (Table 3).

**TABLE 3:** The overall seroprevalence of peste des petits ruminants virus antibodies in both sheep and goat sera in surveyed States of Sudan.

State	Sheep and goat sera					
	Total number tested	%	Number positive	%	Number negative	%
White Nile State	186	50.5	146	78.5	40	21.5
North Kordofan State	19	5.2	18	94.7	1	5.3
South Kordofan State	163	44.3	163	100.0	0	0.0
Total number of sera	368	100.0	327	88.9	41	11.1

### Seroprevalence of peste des petits ruminants virus antibodies in sheep or goat sera in surveyed states of Sudan

Considering the species basis within the surveyed states of Sudan, among sheep, the highest overall seroprevalence of PPRV antibodies was demonstrated in both SK (100%, 142/142) and NK (100%, 10/10) States whereas the least seroprevalence was demonstrated in White Nile State (78.6%, 136/173) (Table 4).

**TABLE 4:** Seroprevalence of peste des petits ruminants virus antibodies in sheep or goat sera in surveyed States of Sudan.

State	Animal species											
	Sheep sera						Goat sera					
	Number tested	%	Number positive	%	Number negative	%	Number tested	%	Number positive	%	Number negative	%
White Nile State	173	53.2	136	78.6	37	21.4	13	30.2	10	76.9	3	23.1
North Kordofan State	10	3.1	10	100.0	0	0.0	9	21.0	8	88.9	1	11.1
South Kordofan State	142	43.7	142	100.0	0	0.0	21	48.8	21	100.0	0	0.0
Total number of sera	325	100.0	288	88.6	37	11.4	43	100.0	39	90.7	4	9.3

Considering the species basis within the surveyed states of Sudan, among goats, the highest overall seroprevalence of PPRV antibodies was demonstrated in SK State (100%, 21/21), followed by NK State (88.9%, 8/9) whereas the least seroprevalence was demonstrated in White Nile State (76.9%, 10/13) (Table 4).

### Seroprevalence of peste des petits ruminants virus antibodies in sheep and goat sera within districts and localities of the White Nile State

Within districts and localities of the White Nile State (Central Sudan), the highest seroprevalence of PPRV antibodies in sheep and goat sera was demonstrated in Gezira Um Jaar, Ed Dueim locality (93.6%, 44/47) followed by Ead Aljam, Ed Dueim locality (76.5%, 78/102) and the least prevalence was presented in Gezira Aba, Rabak locality (64.9%, 24/37) (Table 5).

**TABLE 5:** Seroprevalence of peste des petits ruminants virus antibodies in sheep and goat sera within districts and localities of the White Nile State.

District/locality	Sheep and goat sera						Sheep sera						Goat sera					
	Number tested	%	Number positive	%	Number negative	%	Number tested	%	Number positive	%	Number negative	%	Number tested	%	Number positive	%	Number negative	%
Ead Aljam, Ed Dueim locality	102	54.8	78	76.5	24	23.5	92	53.2	71	77.2	21	22.8	10	76.9	7	70.0	3	30.0
Gezira Um Jaar, Ed Dueim locality	47	25.3	44	93.6	3	6.4	44	25.4	41	93.2	3	6.8	3	23.1	3	100.0	0	0.0
Gezira Aba, Rabak locality	37	19.9	24	64.9	13	35.1	37	21.4	24	64.9	13	35.1	-	-	-	-	-	-
Total number of sera	186	100.0	146	78.5	40	21.5	173	100.0	136	78.6	37	21.4	13	100.0	10	76.9	3	23.1

Considering the species basis, within sheep, the highest seroprevalence of PPRV antibodies was demonstrated in Gezira Um Jaar, Ed Dueim locality (93.2%, 41/44), followed by Ead Aljam, Ed Dueim locality (77.2%, 71/92), and finally the least seroprevalence was presented in Gezira Aba, Rabak locality (64.9%, 24/37) (Table 5). Considering the species basis, within goats, the highest seroprevalence of PPRV antibodies was demonstrated in Gezira Um Jaar, Ed Dueim locality (100%, 3/3) while the least seroprevalence was presented in Ead Aljam, Ed Dueim locality (70%, 7/10) (Table 5).

### Seroprevalence of peste des petits ruminants virus antibodies in sheep and goat sera within districts and localities of Kordofan States

Within districts and localities of NK and SK States (Western Sudan), the highest overall seroprevalence of PPRV antibodies in sheep and goat sera was demonstrated in both Altartar, Eltadamon locality, SK State (100%, 163/163) and in Elobied, Shickan locality, NK State (100%, 10/10), whereas the least seroprevalence was demonstrated in Para, Para locality, NK State (88.9%, 8/9) (Table 6).

**TABLE 6:** Seroprevalence of peste des petits ruminants virus antibodies in sheep and goat sera within districts and localities of Kordofan States.

District/locality	Sheep and goat sera						Sheep sera						Goat sera					
	Number tested	%	Number positive	%	Number negative	%	Number tested	%	Number positive	%	Number negative	%	Number tested	%	Number positive	%	Number negative	%
Para, Para locality	9	4.9	8	88.9	1	11.1	0	0.0	0	0.0	0	0.0	9	30.0	8	88.9	1	11.1
Elobied, Shickan locality	10	5.5	10	100.0	0	0.0	10	6.6	10	100.0	0	0.0	0	0.0	0	0.0	0	0.0
Altartar, Eltadamon locality	163	89.6	163	100.0	0	0.0	142	93.4	142	100.0	0	0.0	21	70.0	21	100.0	0	0.0
Total number of sera	182	100.0	181	99.5	1	0.5	152	100	152	100.0	0	0.0	30	100.0	29	100.0	1	0.0

Considering the species under study, within sheep, the highest seroprevalence of PPRV antibodies was demonstrated in both Altartar, Eltadamon locality, SK State (100%, 142/142) and Elobied, Shickan locality, NK State (100%, 10/10) (Table 6). Considering the species under study, within goats, the highest seroprevalence of PPRV antibodies was demonstrated in Altartar, Eltadamon locality, SK State (100%, 21/21) and the least seroprevalence was detected in Para, Para locality, NK State (88.9%, 8/9) (Table 6).

## Discussion

Since its first recognition at the early 1970s in Sudan (Elhag Ali 1973), outbreaks of PPR were continually reported elsewhere in small ruminants (Osman et al. 2018; Saeed et al. 2010; 2017), camels (Khalafalla et al. 2010; Kwiatek et al. 2011) and lately in gazelles (Asil et al., 2019). The disease has becomes more endemic covering a wide belt in Sudan and the entire African continent (Kwiatek et al. 2011; OIE 2021). In response to several suggestive PPR outbreaks involving sheep and goats in White Nile and Kordofan States during 2018 and 2019, the study aimed to investigate and update information regarding the presence of PPR and for assessment of the serological prevalence of PPRV antibodies in these areas.

Serological surveys performed earlier in the country demonstrated the prevalence of the disease in sheep and goats in the central areas, specifically in Gezira, Khartoum, White Nile, Sinnar and Blue Nile States (Abdalla et al., 2012; Haroun et al. 2002; Ishag et al. 2015; Osman et al. 2009, 2018; Saeed et al. 2010, 2017). Likewise, many sero-surveys performed in sheep and goats demonstrated the prevalence of PPRV antibodies in Western Sudan, in both Kordofan and Darfur States (Abdalla et al., 2012; El Amin & Hassan, 1999; Haroun et al. 2002; Osman et al. 2009; Rasheed 1992; Saeed et al. 2010, 2017; Salih et al., 2014; Shuaib et al. 2014). According to the annual reports of the World Organization for Animal Health (WOAH, formerly OIE), PPR outbreaks were reported regularly in the surveyed states during the last 10 years (OIE World Animal Health Information System [OIE-WAHIS] 2020).

In this study, 88.9% overall seroprevalence of PPRV antibodies was demonstrated when sheep and goat sera were tested by C-ELISA assay. In a similar study conducted during 2016–2017 in North and Central Sudan, lower overall antibodies seroprevalence (80.9%) was demonstrated among sheep and goats using C-ELISA (Osman et al. 2018). The study by Saeed et al. (2017) demonstrated much lower overall seroprevalence of PPRV (49.4%) among different animal species. These results appear much lower than the prevalence achieved in this study.

In this study, goat sera yielded the higher overall seroprevalence of PPRV antibodies (90.7%) compared with sheep sera (88.6%). A relatively lower overall seroprevalence in sheep (84.5%) and goats (66.1%) was demonstrated by Osman et al. (2018). The study by Saeed et al. (2017) demonstrated much lower seroprevalence of PPRV of 67.1% and 48.2% among sheep and goats, respectively. Similarly, 67.2% and 55.6% seroprevalence was achieved from sheep and goats, respectively (Saeed et al. 2010). The results of all previous studies appear much lower than the prevalence achieved in the present study.

Moreover, 100%, 94.7% and 78.5% overall seroprevalences of PPRV antibodies in both sheep and goat sera were demonstrated in SK, NK and White Nile States, respectively. The higher seroprevalence values achieved indicated the wide distribution of the disease in different localities of Kordofan States; however, the incidence of the disease is higher in SK compared with NK; this is possibly due to its increased rate of seasonal animal movement. Due to its location, most of small ruminant herds in NK State are vaccinated regularly during vaccination campaigns organised by the local veterinary authorities, thus may explain the lower seroprevalence values detected. In a similar study conducted during 2016 – 2017, 88.5% and 48.4% overall antibodies seroprevalence was demonstrated among sheep and goats in Gezira and Khartoum States (Central Sudan), respectively (Osman et al. 2018). This result appears much lower than the prevalence achieved in this study.

Among sheep, 100%, 100% and 78.6% overall seroprevalence of PPRV antibodies was demonstrated in SK, NK and White Nile States, respectively. Similarly, among goats, 100%, 88.9% and 76.9% overall seroprevalence of PPRV antibodies was demonstrated in SK, NK and White Nile States, respectively. In contrast, lower overall seroprevalence (74%) was demonstrated among sheep in both Kordofan and Kassala States (Shuaib et al. 2014). Likewise, a much lower overall seroprevalence (68.1%) was detected in sheep sera collected from Darfur States in Western Sudan (Saeed et al. 2017). These results appear much higher compared with 54% seroprevalence value demonstrated during 2008–2009 in White Nile State (Ishag et al. 2015). Of note, a very lower seroprevalence value (39.8%) was demonstrated among unvaccinated sheep and goats in NK State in Sudan (Salih et al. 2014). The results of all previous studies appear much lower than the prevalence achieved in the present study.

In the White Nile State (Central Sudan), 93.6%, 76.5% and 64.9% seroprevalence of PPRV antibodies in small ruminants sera was demonstrated in Gezira Um Jaar, Ed Dueim locality; Ead Aljam, Ed Dueim locality; and Gezira Aba, Rabak locality, respectively. Among sheep, 93.2%, 77.2% and 64.9% seroprevalence of PPRV antibodies was demonstrated in Gezira Um Jaar, Ed Dueim locality; Ead Aljam, Ed Dueim locality; and Gezira Aba, Rabak locality, respectively. Among goats, 100% and 70% seroprevalence of PPRV antibodies was demonstrated in Gezira Um Jaar, Ed Dueim locality and Ead Aljam, Ed Dueim locality, respectively. Generally, slightly higher seroprevalence values, among small ruminants, were achieved in this study than previously demonstrated. The reason for the higher seroprevalence among sheep than in goats is that higher fatalities were observed among goats.

In Kordofan States (Western Sudan), 100%, 100% and 88.9% seroprevalence of PPRV antibodies in small ruminants sera was demonstrated in Altartar, Eltadamon locality, SK State; Elobied, Shickan locality, NK State; and Para, Para locality, NK State, respectively. Among sheep, 100% and 100% seroprevalence of PPRV antibodies was demonstrated in Altartar, Eltadamon locality, SK State and Elobied, Shickan locality, NK State, respectively. Among goats, 100% and 88.9% seroprevalence of PPRV antibodies was demonstrated in Altartar, Eltadamon locality, SK State and Para, Para locality, NK State, respectively. In contrary, lower seroprevalence of PPRV (74.5%) was demonstrated among sheep in NK State (Shuaib et al. 2014). In other study, lower seroprevalence (58.3%) in sheep and (38.8%) in goats was demonstrated in Kordofan States during 2008–2010 (Saeed et al. 2017). These results appear much lower than seroprevalence values achieved in this study.

The findings of the study indicated the occurrence of PPR in mixed herds of sheep and goats in the investigated areas. The higher seroprevalence values achieved, although small ruminants had never been vaccinated against PPR, indicated the wide exposure of these animals to PPRV. Our suspicion of PPRV infection, due to appearance of typical clinical signs of PPR in infected small ruminants in areas of outbreaks, was confirmed by laboratory diagnosis using IC-ELISA. The continual presence of PPR outbreaks in different areas in Sudan might influence the currently established global eradication programme of the disease which was launched by the WOAH, formerly OIE, and the Food and Agriculture Organization (FAO). Animal herds in these areas have a special pattern of seasonal movement towards the South at the beginning of the hot summer season and backwards at the beginning of autumn. Some animals may cross the country borders entering the neighbouring countries such as the Republic of South Sudan where it is staying there for many months and escape vaccination which takes place during this period. In fact, PPR is endemic in all African countries bordering Sudan. Sharing grazing areas with infected small ruminants and other animal species in neighbouring countries could contribute to appearance of outbreaks with new PPRV strains or lineages via contact with infected small ruminants. To prevent spread of the disease and presence of new outbreaks, it is recommended that the vaccination campaigns must reach all herd sectors.
